# Going beyond lifestyle factors

**DOI:** 10.7554/eLife.70548

**Published:** 2021-06-24

**Authors:** Milagros Ruiz

**Affiliations:** Research Department of Epidemiology and Public Health, University College LondonLondonUnited Kingdom

**Keywords:** social determinants of health, biopsychosocial, socio-economic status, hierarchy, mismatch, health, Human

## Abstract

Wealth and inequality impact blood pressure in a population with the lowest risk of heart disease in the world.

**Related research article** Jaeggi AV, Blackwell AD, von Rueden C, Trumble BC, Stieglitz J, Garcia AR, Kraft T, Beheim BA, Hooper PL, Kaplan H, Gurven MD. 2021. Do wealth and inequality associate with health in a small-scale subsistence society?. *eLife* **10**:e59437. doi: 10.7554/eLife.59437

The idea of a ‘social ladder’ may be metaphorical, but actual and perceived societal ranking have real consequences for the health of an individual (Adler et al., 2000). How social structure influences health and disease is overwhelmingly studied in high-income countries, where coronary heart disease (CHD for short) is the leading cause of death (Institute for Health Metrics and Evaluation, 2019).

In these societies, the relationship between an individual’s social position and their CHD risk is astonishingly consistent, with disadvantaged populations being more likely to suffer from the disease and to die from it (Schultz et al., 2018). Despite the clarity of this evidence, the public health workforce has not yet reached a unified consensus on why these inequalities occur, and what can be done to reduce them (Marmot, 2004).

Lifestyle factors such as diet, sedentarism or smoking, and their ensuing effects like hypertension, only partially account for the excess burden of CHD in disadvantaged groups (Schultz et al., 2018). Yet primary prevention efforts appear to focus on these health behaviours over other factors linked to social inequality. In fact, targeting lifestyle alone is likely to exacerbate inequalities in post-industrial societies (Marmot, 2004).

Investigating inequalities in populations that have not adopted Western diets and activity levels – a challenging undertaking given the proliferation of this lifestyle worldwide – could be a way to confront the underlying assumption that behavioural differences are responsible for the observed inequality in CHD (Kopp, 2019). Now, in eLife, Adrian Jaeggi (University of Zurich and Emory University), Aaron Blackwell (Washington State University) and co-workers based in the United States, France and Germany report the most comprehensive study on social structure and health in a pre-industrial society in the Bolivian Amazon known as the Tsimane (Jaeggi et al., 2021). 

This population relies on subsistence farming supplemented by hunter-gatherer practices, resulting in an extremely physically active life and a diet that is rich in fibres and micronutrients. In turn, they have remarkably modest rates of obesity and hypertension, and the lowest prevalence of biological markers for poor artery health ever recorded around the world (Pontzer et al., 2018). Thus, any putative relationship between social position and heart health is unlikely to be the result of differences in health behaviour.

Overall, Jaeggi et al. discovered consistent links between wealth-related circumstances and blood pressure in the Tsimane: the poorer the individual, the higher their blood pressure. In people over the age of 15, the pressure on artery walls during and between heartbeats was lower in those with higher household wealth, that is, those with more common household assets: this can include traditional goods made from local organic materials, industrially produced items acquired through trade or purchase, and livestock. The researchers also investigated the association between wealth inequality and overall health in several geographically separated communities – defined as clusters of households connected through kin networks that produce or consume food together. They found that communities with greater inequality between rich and poor members had higher blood pressure.

Most Tsimane have normal blood pressure. This means that associations between wealth and individual blood pressure within communities, or between wealth inequality and overall blood pressure across communities both capture variations below a clinically significant level (Jaeggi et al., 2021; Pontzer et al., 2018). However, these findings are not inconsequential: in post-industrial societies, small reductions in blood pressure in the overall population have proved effective in lowering CHD incidence (Cook et al., 1995).

If no members of the Tsimane population live an unhealthy lifestyle, and if they all have little to no access to healthcare, then what drives higher blood pressure in poorer adults and in more unequal communities? Psychosocial mechanisms and pathways to poor health may provide an answer, drawing on how feelings which result from inequality, domination, or subordination may directly alter biological processes (Bartley, 2017). Social hierarchies, maintained by societal arrangements of power, lead to disadvantaged populations being disproportionally exposed to psychosocial stressors such as lack of community support, low control and autonomy, and an imbalance between effort and reward. In turn, psychosocial stress can have a severe impact on the body, triggering a sustained fight or flight response and altering the hormone system that controls biological reactions to stress (Bartley, 2017; Jaeggi et al., 2021).

Jaeggi et al. therefore tested how psychosocial factors related to unequal wealth and wealth distribution may have influenced feelings and interactions among the Tsimane (e.g., depression, social conflicts), or altered their body chemistry (e.g., the level of the stress hormone cortisol in urine). The analyses highlighted a weak connection between these factors and increased levels of blood pressure in individuals who possess less wealth or are from more unequal communities. However, this link may only be weakly supported by the analyses because the markers used could have insufficiently measured psychosocial stress. It may therefore be worth also examining whether a pathway can be identified when looking at C-reactive protein, an inflammatory biomarker for blood pressure which is relatively elevated in the Tsimane population (Pontzer et al., 2018). Yet, detecting these small effects in such a healthy society requires a large sample size, and psychosocial markers were only collected in a subset of participants with blood pressure data: thus, it is more likely that the analyses were underpowered.

The Tsimane face growing exposure to psychosocial stress as contact with ethnic majority groups increase, and their economy becomes more integrated. These developments urge researchers to explore individual-level and macro-level mechanisms for health inequality in the Tsimane, and remind us, once again, to look beyond lifestyle when tackling public health problems.
